# Beyond biology: the impact of marital status on survival of patients with adrenocortical carcinoma

**DOI:** 10.1590/S1677-5538.IBJU.2014.0348

**Published:** 2015

**Authors:** Zachary Klaassen, Lael Reinstatler, Martha K. Terris, Willie Underwood, Kelvin A. Moses

**Affiliations:** 1 Department of Surgery, Section of Urology, Medical College of Georgia, Georgia Regents University, Augusta, GA, USA; 2 Department of Urology, Roswell Park Cancer Institute, Buffalo, NY, USA

**Keywords:** Adrenocortical Carcinoma, Marital Status, Social Class, Survival, Disease

## Abstract

**Purpose:**

To analyze the association of marital status and survival of patients with ACC using a population-based database.

**Material and Methods:**

Patients with ACC were abstracted from the Surveillance Epidemiology and End Results (SEER) database from 1988-2010 (n=1271). Variables included marital status (married vs single/divorced/widowed (SDW)), gender, age, race, tumor (T) and node (N) classification, receipt of surgery, and SEER stage. Statistical analysis was performed using Cox proportional hazard models to generate hazard ratios and 95% confidence intervals.

**Results:**

There were 728 (57.3%) females and median age was 56 years (IQR 44-66). Patients who were alive were more frequently married (65.6% vs 61.6%, p=0.008), female (61.1% vs 58.0%, p=0.001), younger (median 51 vs 57 years, p=0.0001), submitted to adrenalectomy (88.6% vs 63.8%, p<0.0001), and more favorable SEER stage (localized-64.9% vs 29.9%; regional–25.1% vs 30.1%; distant 4.8% vs 31.5%, p<0.0001) compared to patients dead of disease (DOD). On multivariable analysis, factors significantly associated with all-cause mortality were SDW status (HR 1.28, 95% CI 1.091.51), age, non-operative management, and N+ disease. Risk factors for disease-specific mortality included SDW status (HR 1.30, 95% CI 1.07-1.56), age, non-operative management, T-classification, and N+ disease.

**Conclusions:**

Marital status is significantly associated with survival in patients with ACC. Our results suggest that the decreased survival seen among SDW individuals highlights an area for further research and needed intervention to reduce disparity.

## INTRODUCTION

Adrenocortical carcinoma (ACC) is a rare malignancy with a reported incidence of 0.5-2 per million, a recurrence rate of 60-80%, and 5-year overall survival of 20-47% (1, 2). Despite advances in imaging and treatment regimens over the past 20 years, survival outcomes in patients with ACC continue to remain poor. Therefore, clinicians must seek additional factors to optimize outcomes in this select group of patients.

The effect of marital status on disease specific survival (DSS) in patients with cancer has been reported across several malignancies, although the reason for a survival benefit provided by marriage has not been completely elucidated (3–9). In a recent study analyzing the impact of marital status on the 10 leading causes of cancer-related death in the US, Aizer et al. found that single-divorced-widowed (SDW) patients were at greater risk of presentation with metastatic disease, under treatment and cancer specific mortality (3). These results suggest that SDW patients with malignancy represent an at-risk population that may benefit from structured support and intervention.

Apart from the known risk factors that impact survival such as TNM classification, we sought to identify other significant factors specific to survival outcomes. Given the poor survival associated with ACC and paucity of literature reporting the effect of socioeconomic variables on survival in these patients, the objective of this study was to assess the impact of marital status on overall survival (OS) and DSS in patients with ACC. Furthermore, we sought to identify other non-clinical or pathologic factors that may be associated with greater risk of mortality. Our hypothesis was that SDW patients and patients with poorer socioeconomic status (SES) would have worse OS and DSS compared to married patients and those with more favorable SES.

## MATERIALS AND METHODS

Study Population: The study cohort consisted of patients from all 18 registries comprising the Surveillance, Epidemiology and End Results (SEER) database from 1988-2010. The SEER database reports cancer specific outcomes from specific geographic areas representing 28% of the US population (10). Patients ≥18 years of age with ACC were identified in the SEER database utilizing the primary site codes C74.0 and C74.9, and International Classification of Diseases for Oncology, 9^th^ edition (ICD-9) code 1940 for a study cohort of 1271 patients. Patients were divided into three groups (alive, dead of disease (DOD) and dead of other causes (DOC)).

Description of Covariates: Demographic variables of interest included marital status (married vs single/divorced/widowed (SDW)), gender, age at diagnosis, race (African American vs Caucasian vs Hispanic vs other), SEER registry, and median census county data for educational attainment (<9th grade vs <high school vs >Bachelor degree), poverty level, % foreign born, % unemployed, and household income. Clinical and pathologic variables included receipt of surgery (adrenalectomy vs none vs other), laterality (left vs right vs bilateral), American Joint Committee on Cancer (AJCC) 7^th^ edition tumor (T) and node (N) classification, metastasis (yes/no), SEER stage (localized vs regional vs distant vs unstaged), and median OS (SEER survival data–censoring date September 10, 2013).

### Statistical analysis

Descriptive statistics for demographic and clinicopathological variable comparisons was performed using t-test and Chi square test. Survival estimates were calculated using the Kaplan-Meier method for OS and DSS by marital status, gender, age at diagnosis, race, T-classification, and N-classification. Cox proportional hazard analysis was performed to generated hazard ratios for risk factors of mortality. The model was constructed and analyses were performed using backward selection, removing all insignificant variables until the best-fit model was achieved. In this model, T-classification and N-classification were adjusted for, while SEER stage was not adjusted for in order to refrain from including confounding variables. Statistical analyses were performed using SAS 9.3 (SAS Institute, Cary, NC). All tests were two-sided and with a statistical significance set at p<0.05.

## RESULTS

### 

#### Population Demographics

There were 728 females (57.3%) and 543 males (42.7%) with a median age of 56 years (IQR 44-66). There were 422 patients who were alive (33.2%), 685 patients (53.9%) DOD and 164 patients (12.9%) DOC (Table-1). A significantly higher percentage of alive patients were married (65.6% vs 61.6% vs 51.8%; p=0.008), female (61.1% vs 58.0% vs 44.5%; p=0.001) and younger at diagnosis (median 51 years vs 57 years vs 65 years; p<0.001) compared to those DOD and DOC, respectively. There was no significant difference for educational status, poverty/ income, foreign born status, and unemployment between the groups.

**Table 1:** Demographics of 1271 patients with adrenocortical carcinoma.

Variable		Alive	Dead of Disease	Dead other Causes	p-value
Patients, n (%)		422 (33.2)	685 (53.9)	164 (12.9)	
**Marital status, n (%)**					0.008
	Married	277 (65.6)	422 (61.6)	85 (51.8)	
	SDW	134 (31.8)	241 (35.2)	74 (45.1)	
	Unknown	11 (2.6)	22 (3.2)	5 (3.1)	
**Gender, n (%)**					0.001
	Male	164 (38.9)	288 (42.0)	91 (55.5)	
	Female	258 (61.1)	397 (58.0)	73 (44.5)	
**Median age, years (IqR)**		51 (42, 61)	57 (44, 66)	65 (56, 74)	<0.001
**Race, n= (%)**					0.44
	Caucasian	320 (75.8)	533 (77.8)	121 (73.8)	
	Hispanic	40 (9.5)	64 (9.4)	19 (11.5)	
	AAM	27 (6.4)	42 (6.1)	16 (9.8)	
	Other	35 (8.3)	46 (6.7)	8 (4.9)	
Median <9^th^ Grade Education, % (IQR)		5.7 (3.9, 8.9)	5.9 (3.7, 9.9)	5.9 (3.8, 8.9)	0.88
Median <High School Education, % (IQR)		13.9 (10.0, 20.1)	14 (9.9, 20.3)	13.5 (11.3, 18.9)	0.78
Median >Bachelor Degree, % (IQR)		29.2 (21.6, 35.6)	29.2 (23.2, 39.6)	30.1 (23.3, 38.4)	0.05
Median <Poverty, % (IQR)		13.4 (10.5, 16.3)	12.3 (10.2, 16.3)	12.2 (9.9, 16.3)	0.56
Median Foreign Born, % (IQR)		15.4 (6.9, 28.8)	17.8 (7.7, 30.5)	16.3 (9.4, 29.4)	0.38
Median Unemployed, % (IQR)		9.1 (7.9, 9.8)	9.2 (7.5, 9.8)	9.2 (7.7, 9.8)	0.85
Median Household Income, % (IQR)		56,550 (48,340, 67,010)	57,580 (51,770, 70,570)	58,820 (54,090, 70,570)	0.08

**SDW =** single/divorced/widowed; **IQR =** interquartile range; **AAM =** African American; **SEER =** Surveillance Epidemiology and End Results

#### Clinicopathologic Analysis

**Figure 1:**
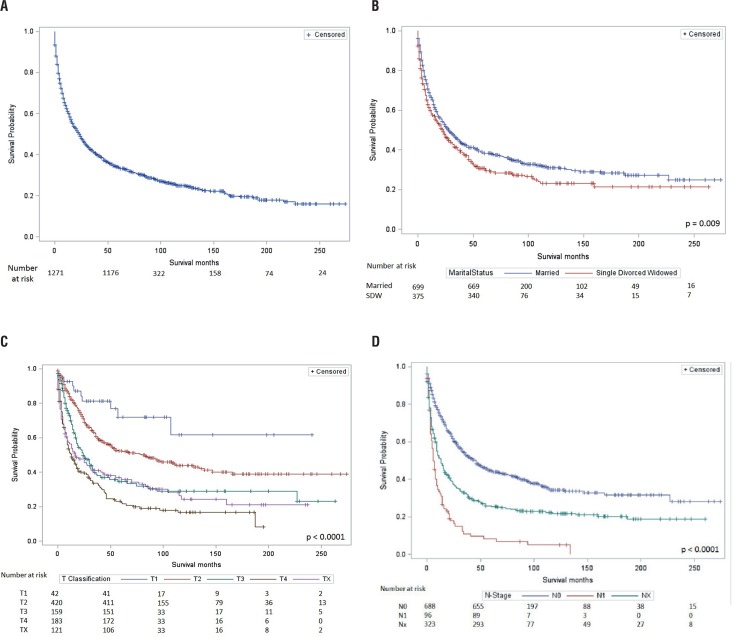
Kaplan-Meier survival estimates for (A) overall survival; (B) disease-specific survival by marital status (p=0.009); (C) disease-specific survival by T-classification (p<0.0001), and (D) disease-specific survival by n-classification (p<0.0001).

Patients who were alive had a higher frequency of adrenalectomy (88.6% vs 63.8% vs 67.7%; p<0.0001) compared to patients DOD or DOC, respectively (Table-2). Furthermore, patients who were alive had more favorable T classification (p<0.0001), more favorable N classification (p<0.0001), less frequency of metastatic disease (1.7% vs 25.4% vs 17.1%; p<0.0001), and have more favorable SEER stage (p<0.0001) compared to patients DOD or DOC. Median survival was 9 months (IQR 3-24) for patients DOD and 10 months (IQR 1-51) for patients DOC (Figure-1A). DSS was statistically significantly associated with marital status (Figure-1B, p=0.009), age at diagnosis (decade) (Figure-1D, p<0.0001), T-classification (Figure-1E, p<0.0001), and N-classification (Figure-1F, p<0.0001), but was not associated with gender (Figure-1C, p=0.18).

**Table 2:** Clinical and pathologic variables of 1271 patients with adrenocortical carcinoma.

Variable		Alive	Dead of Disease	Dead other Causes	p-value
**Surgical Approach, n (%)**					<0.0001
	Adrenalectomy	374 (88.6)	437 (63.8)	111 (67.7)	
	None	36 (8.5)	212 (30.9)	41 (25.0)	
	Other	11 (2.6)	30 (4.4)	9 (5.5)	
**Laterality, n (%)**					0.08
	Left	222 (52.6)	347 (50.7)	96 (58.5)	
	Right	190 (45.0)	300 (43.8)	61 (37.2)	
	Bilateral	1 (0.3)	8 (1.1)	1 (0.6)	
**T Classification, n (%)**					<0.0001
	TX	39 (9.3)	82 (12.0)	30 (18.3)	
	T0	1 (0.2)	0	0	
	T1	32 (7.6)	10 (1.5)	14 (8.5)	
	T2	228 (54.0)	192 (28.0)	57 (34.8)	
	T3	68 (16.1)	91 (13.3)	18 (11.0)	
	T4	47 (11.1)	136 (19.8)	31 (18.9)	
**N Classification, n (%)**					<0.0001
	NX	79 (18.7)	244 (35.6)	58 (35.4)	
	N0	332 (78.7)	356 (52.0)	89 (54.2)	
	N1	11 (2.6)	85 (12.4)	17 (10.4)	
Metastasis, n (%)		7 (1.7)	174 (25.4)	14 (8.5)	<0.0001
**SEER Stage, n (%)**					<0.0001
	Localized	274 (64.9)	205 (29.9)	77 (46.9)	
	Regional	106 (25.1)	206 (30.1)	40 (24.4)	
	Distant	20 (4.8)	216 (31.5)	28 (17.1)	
	Unstaged	22 (5.2)	58 (8.5)	19 (11.6)	
**Median Survival**, months (IQR)		53 (17, 112)	9 (3, 24)	10 (1, 51)	<0.0001

**SEER =** Surveillance Epidemiology and End Results; **IQR =** interquartile range

#### Risk Factors for Mortality

On multivariable analysis, significant factors associated with increased risk of all-cause mortality were SDW status (HR 1.28, 95% CI 1.09-1.51), older age (HR 1.43, 95% CI 1.31-1.55), % <9^th^ grade education (HR 1.06, 95% CI 1.001.13), non-operative management (HR 3.18, 95% CI 2.57-3.95), T-classification (TX vs T1–HR 1.78, 95% CI 1.12-2.82; T3 vs T1–HR 2.28, 95% CI 1.453.60; T4 vs T1–HR 2.58, 95% CI 1.66-4.03), and N+ disease (HR 2.27, 95% CI 1.74-2.97). SDW status was also a significant risk factor for disease-specific mortality (HR 1.30, 95% CI 1.07-1.56), as did older age (HR 1.46, 95% CI 1.32-1.61), non-operative management (HR 3.56, 95% CI 2.804.52), T-classification (TX vs T1–HR 2.58, 95% CI 1.30-5.13; T2 vs T1–HR 2.19, 95% CI 1.14-4.22; T3 vs T1–HR 3.66, 95% CI 1.87-7.14; T4 vs T1– HR 3.97, 95% CI 2.05-7.69), and N+ disease (HR 2.37, 95% CI 1.76-3.19). Gender was included in the model and additionally in the Kaplan-Meier analysis and there was no significant difference in outcomes on multivariate analysis.

## DISCUSSION

This population-based study analyzing factors associated with mortality in patients with ACC demonstrates that SDW patients have significantly worse all cause and cancer specific mortality compared to married patients. Furthermore, age was also associated with mortality in addition to poor staging characteristics. Although male patients had worse outcomes on univariate analysis, gender did not retain significance as a risk factor on multivariable analysis. In contrast to many studies in other malignancies, there was no significance found for ethnicity or socioeconomic status on outcomes. The importance of this study is that it is the first to show the effect of marital status on mortality outcomes in patients with ACC.

The survival benefit associated with marital status has been described in other urologic malignancies, including bladder, prostate, penile and testis cancer (4–9). Gore and colleagues demonstrated a clear survival benefit among married patients undergoing radical cystectomy identified within the SEER database from 1973-2000 compared to SDW patients (6). This positive effect was an independently significant factor even when controlling for pathological factors, gender and race. Sammon et al. analyzed 14,859 patients undergoing radical cystectomy (RC) between 1988 and 2006 and found that never-married males had a higher rate of non-organ confined disease at RC, a trend not observed in never-married females (4). SDW men and women also displayed a higher rate of all-cause mortality and disease specific mortality. In an analysis of the SEER database from 1988-2006, Abdollah et al. identified 163,697 men undergoing radical prostatectomy (RP) with organ confined prostate cancer (7). They found that men who were SDW had more advanced stage at RP and higher cause-specific and all-cause mortality compared to married men. The same group assessed the effect of marital status on OS and cancer-specific mortality for patients with squamous cell carcinoma of the penis (8). Between 1988 and 2006, they identified 1,844 patients with squamous cell carcinoma of the penis and found that unmarried men had a 1.5-fold higher risk for locally advanced disease at surgery and 1.3-fold higher risk of overall mortality. Interestingly, unmarried men did not have an increased of cause-specific mortality in this cohort.

**Table 3:** Cox proportional analysis of risk factors for mortality in 1271 patients with adrenocortical carcinoma.

		Overall Mortality HR (95% CI)	p-value	Cancer Specific Mortality HR (95% CI)	p-value
**Marital status**	Married	Ref		Ref	
	SDW	1.28 (1.09-1.51)	0.003	1.30 (1.07-1.56)	0.007
**Gender**	Female	Ref		Ref	
	Male	1.17 (1.00-1.38)	0.049	1.02 (0.85-1.22)	0.84
**Age** #	Per decade	1.43 (1.31-1.55)	<0.0001	1.46 (1.32-1.61)	<0.0001
**Race**	White	Ref		Ref	
	African-American	1.29 (0.95-1.77)	0.11	1.17 (0.80-1.72)	0.42
	Hispanic	1.22 (0.92-1.61)	0.18	1.12 (0.80-1.56)	0.50
	Other	1.07 (0.76-1.50)	0.71	0.97 (0.66-1.42)	0.85
**%<9^th^ Grade Education**	Per 1% change	1.06 (1.00-1.13)	0.039	1.07 (0.99-1.14)	0.055
**%< High school Education**	Per 1% change	0.96 (0.93-0.99)	0.042	0.96 (0.92-0.99)	0.038
**Laterality**	Left	Ref		Ref	
	Right	0.89 (0.76-1.04)	0.15	0.87 (0.73-1.05)	0.15
	Bilateral	0.96 (0.42-2.19)	0.92	0.99 (0.43-2.31)	0.99
**Surgery**	Adrenalectomy	Ref		Ref	
	None	3.18 (2.57-3.95)	<0.0001	3.56 (2.80-4.52)	<0.0001
	Other	1.11 (0.73-1.70)	0.62	1.17 (0.70-1.98)	0.55
**T Classification**	T1	Ref		Ref	
	TX	1.78 (1.12-2.82)	0.014	2.58 (1.30-5.13)	0.007
	T2	1.44 (0.93-2.22)	0.10	2.19 (1.14-4.22)	0.018
	T3	2.28 (1.45-3.60)	0.0004	3.66 (1.87-7.14)	0.0001
	T4	2.58 (1.66-4.03)	<0.0001	3.97 (2.05-7.69)	<0.0001
**N Classification**	N0	Ref		Ref	
	NX	0.92 (0.76-1.11)	0.37	0.96 (0.77-1.20)	0.73
	N1	2.27 (1.74-2.97)	<0.0001	2.37 (1.76-3.19)	<0.0001

**HR =** hazard ratio; **SEER =** Surveillance, Epidemiology and End Results

# Decades: 18-59, 60-69, 70-79, 80+

The benefit of marriage in patients with cancer likely represents a stable social construct, although the reasoning is subjective and the effect is likely multifactorial. For example, being married may reflect better access to healthcare compared to unmarried patients. However, better access to healthcare cannot be the total explanation because poor socioeconomic status still adversely affects outcomes in countries that have universal healthcare (3, 11). Psychosocial factors associated with being married may be influential as well. Married patients may be encouraged by their spouses to seek medical attention for worrisome symptoms, seek definitive treatment for conditions, and adhere to prescribed treatment regimens with the encouragement of a supportive spouse (3, 12, 13). The effect of this bond is speculative, however it may be inferred that this relationship may improve adherence to treatment regimens and rigorous follow-up required of cancer patients. The diagnosis of cancer may illicit distress and subsequently depression. By having a supportive partner, married patients have the ability to share the emotional burden of a cancer diagnosis with their spouse (14). Ultimately, patients without a support system associated with marriage may be at risk for poor outcomes and may require additional effort in order to maximize excellent clinical outcomes.

Although the overall and cancer specific mortality rates for patients with ACC is dismal even in married patients, the role of clinicians and multidisciplinary teams is to recognize these disparities in outcomes for the SDW patient and provide avenues to improve survival for these patients to the level of the married patient. Furthermore, interventions to improve outcomes for the SDW patient may prove to be cost-effective in the overall healthcare structure and deliverability of care. Previous studies outside of the urologic community have assessed the impact of promoting support mechanisms in oncology patients (15, 16). In a study from the Massachusetts General Hospital, investigators randomly assigned 107 patients with metastatic non-small-cell lung cancer to receive either early palliative care in conjunction with standard oncologic care or standard oncologic care alone (15). The authors found that patients receiving early palliative care had improved quality of life and mood, required less aggressive end-of-life care and ultimately had longer median survival (11.6 months vs. 8.9 months, p=0.02) compared to the control group. Aoun et al. randomized 26 palliative care patients living alone to having additional care-aid hours in their home and found that these patients had improved quality of life, preservation of self-dignity, ease of burden of everyday living, and reduced loneliness and isolation (16). Although similar studies have not been performed in married vs. SDW patients, particularly for the dismal prognosis associated with ACC, implementing comparable measures to the SDW population should be an area of further research endeavors.

There are some limitations that must be considered when interpreting the data. First, as with any retrospective analysis of a large administrative database, SEER does not provide sufficient granularity to predict causal factors specifically related to marital status and survival such as length of marriage, marital satisfaction, unmarried co-habitational relationships, or long-term homosexual relationships. Secondly, the SEER database does not include details that may impact marital status such as the quality of the relationship, length of marriage, or health of the spouse. Furthermore, patients who are SDW may have a support system equivalent to that often associated with marriage (eg. extended family, coworkers, and friends). Finally, the SEER database does not contain information related to patient comorbidities, which may be an unaccounted confounding factor in the causal association between SDW patients and inferior survival outcomes, nor does it contain information regarding receipt of chemotherapy, which would be important to know for patients with high stage disease or recurrence. The major strength of this study is that it is the first to identify the impact of marital status on survival in ACC. These results suggest that a properly designed and implemented intervention for SDW patients may have a modest impact on this and other malignancies.

## CONCLUSIONS

ACC is a disease with an overall poor prognosis due to aggressive biological behavior. SDW status is associated with poorer survival in patients with ACC, suggesting that the decreased survival seen among SDW individuals in other urologic malignancies may also be relevant for patients with ACC. Health care providers caring for unmarried patients with ACC should be aware of the poorer outcomes in these patients, highlighting an area for further research and implementation of improved support systems to reduce this disparity and improve their survival to that of married patients.

## Abbreviations

ACC: = adrenocortical carcinoma
SDW: = single/divorced/widowed
DSS: = disease specific survival
SES: = socioeconomic status
SEER: = Surveillance, Epidemiology and End Result
DOD: = dead of disease
DOC: = dead of other causes
